# Estimation and Validation of Dietary Nitrate and Nitrite Intake in Iranian Population

**Published:** 2019-01

**Authors:** Zahra BAHADORAN, Asghar GHASEMI, Parvin MIRMIRAN, Yadollah MEHRABI, Fereidoun AZIZI, Farzad HADAEGH

**Affiliations:** 1. Nutrition and Endocrine Research Center, Research Institute for Endocrine Sciences, Shahid Beheshti University of Medical Sciences, Tehran, Iran; 2. Endocrine Physiology Research Center, Research Institute for Endocrine Sciences, Shahid Beheshti University of Medical Sciences, Tehran, Iran; 3. Department of Epidemiology, School of Public Health, Shahid Beheshti University of Medical Sciences, Tehran, Iran; 4. Endocrine Research Center, Research Institute for Endocrine Sciences, Shahid Beheshti University of Medical Sciences, Tehran, Iran; 5. Prevention of Metabolic Disorders Research Center, Research Institute for Endocrine Sciences, Shahid Beheshti University of Medical Sciences, Tehran, Iran

**Keywords:** Nitrate, Nitrite, Diet, Food frequency questionnaire

## Abstract

**Background:**

The aim of this study was calibration of a nitrate (NO_3_)/nitrite (NO_2_) database for estimated its dietary intakes.

**Methods:**

Overall, 250 healthy Tehranian adults were assessed in 2015 for dietary intakes of NO_3_ and NO_2_ and its serum and urine concentration. Food composition values for NO_3_ and NO_2_ were derived from a recent survey conducted on frequently consumed food items among Iranians. The correlation of dietary intakes of NO_3_/NO_2_ and its urinary and serum values was evaluated.

**Results:**

Mean (±SD) intakes of dietary NO_3_ and NO_2_ were 505±160 and 7.7±2.2 mg/d, respectively. The correlation coefficient of intake and urinary NO_3_ was 0.83 (95% CI=0.56–0.91) and 0.57 (95% CI=0.49–0.67) in men and women, respectively. A moderate agreement was also observed between NO_2_ intake and its urinary levels (*r*=0.27, 95% CI=0.13–0.37, and 0.29, 95% CI=0.17–0.41, in men and women, respectively).

**Conclusion:**

Using a national database of NO_3_ and NO_2_ content of food items along with a valid food frequency questionnaire could provide a valid estimation of dietary intakes of NO_3_ in the target population.

## Introduction

Inorganic nitrate (NO_3_) and nitrite (NO_2_) are compounds occurring in both natural and industrially processed foods; vegetables and drinking water are major sources of dietary NO_3_ and vegetables especially green leafy vegetables, including lettuce and spinach, cabbage, rocket, red beet-root, and radish contribute approximately 80%–95% of the dietary intake of NO_3_, whereas major dietary NO_2_ intakes are usually from processed meat and animal food products (1, 2). Historically, there has been a long-term concern regarding adverse effects of dietary NO_3_ and NO_2_ due to its potential endogenous conversion to nitrosa-mines, and some acute and chronic toxicities such as methemoglobinemia, thyroid disorders and carcinogenesis (3–5). In contrast, recent research punctuates that dietary NO_3_/NO_2_ may induce several beneficial effects especially on cardiovascular system and metabolic pathways (6–8). There is also some evidence to support consideration inorganic NO_3_/NO_2_ as dietary nutrients (9). Despite growing agreement regarding an urgent need to clarify longitudinal risk-benefit assessment of NO_3_ and NO_2_ intakes on human health in the framework of population-based studies, there is a huge gap of knowledge in this regard (5, 10–12). Valid estimation of NO_3_ and NO_2_ exposure from diets seems to be a main challenging issue and important barrier to such investigations (13). Unlike other nutrients and food components, there is no valid applicable and comprehensive database such as the US Department of Agriculture (USDA) food composition table (FCT) for NO_3_ and NO_2_; substantial efforts have therefore been made during two past decades in several countries to develop such database (14–21).

Due to great variations in the NO_3_ and NO_2_ contents of foods, estimated dietary NO_3_ and NO_2_ intakes from FFQ needs to be confirmed by relevant serum or urine biomarkers (22). Serum and urinary NO_3_ and NO_2_ concentrations may be considered as reliable biomarkers of exogenous exposure to NO_3_ and NO_2_ and the majority of the urinary nitrate can be accounted for by dietary sources (23, 24); however, some confounders such as endogenous nitric oxide production, biological factors, physical activity, smoking, and some pathologic conditions may affect circulating levels of NO_3_ and NO_2_, an issue that need to be considered in the analysis (23).

To provide the opportunities for assessment of health related outcomes of dietary exposure to NO_3_ and NO_2_ in the framework of epidemiological studies and prospective cohorts, we recently developed a database for NO_3_ and NO_2_ contents of commonly consumed food items among Iranians (25).

Here, we described the performance of the database for estimation of NO_3_ and NO_2_, intake, in a representative Iranian adult population from the Tehran Lipid and Glucose Study, and evaluate the estimated values in relation to serum and urinary NO_3_ and NO_2_ levels.

## Methods

### Study population

The current calibration study was conducted in the subset of cohort members of the Tehran Lipid and Glucose Study (TLGS), an ongoing community-based prospective study being conducted to investigate and prevent noncommunicable diseases, in a representative sample in the district 13 of Tehran, the capital city of Iran (26). Briefly, healthy non-smoker adults, aged 20–70 yr, free of type 2 diabetes, hypertension, coronary heart disease and renal dysfunction, were consecutively recruited from the TLGS, between Jul to Oct 2015 (26). Participants who had under- or over-report of total energy intakes (<800 kcal/d or >4200 kcal/d) or had specific diet (including dietary recommendations for HTN, hyperlipidemia or diabetes) were excluded and final analysis was conducted on 251 men and women.

Written informed consents were obtained from all participants, and the study protocol was approved by the Ethics Research Council of the Research Institute for Endocrine Sciences, Shahid Beheshti University of Medical Sciences, in accordance with the Helsinki Declaration of 1975 as revised in 1983.

### Demographic and anthropometric measures

Trained interviewers collected information using standard questionnaires. Detailed measurements of variables in TLGS have been reported elsewhere (27). Weight was measured to the nearest 100 gr using digital scales, while the subjects were minimally clothed, without shoes. Height was measured to the nearest 0.5 cm, in a standing position without shoes, using a tape meter. Body mass index (BMI) was calculated as weight (kg) divided by square of the height (m^2^). Waist circumference (WC) was measured to the nearest 0.1 cm, midway between the lower border of the ribs and the iliac crest at the widest portion, over light clothing, using a soft measuring tape, without any pressure to the body.

For measurements of systolic (SBP) and diastolic blood pressure (DBP), after a 15-min rest in the sitting position, two measurements of blood pressure were taken on the right arm, during a standardized mercury sphygmomanometer; the mean of the two measurements was considered as the participant's blood pressure.

### Biochemical measures

Blood and urine samples were taken after 12–14 h fasting from all study participants. Serum creatinine (Cr) levels were assayed by kinetic colorimetric Jaffe; the sensitivity of the assay was 0.2 mg/dL (range, 18–1330 μmol/L (0.2–15 mg/dL) (28). Serum and urine NO_3_ and NO_2_ concentrations were measured by a rapid, simple spectrophotometric method for simultaneous detection of nitrate and nitrite (29). This method has been previously validated in our laboratory and a review paper regarding serum nitric oxide metabolites (NO_x_) measurement has been published by our group (30, 31). Inter- and Intra-assay coefficients of variations of the assays were 5.2% and 4.4%, respectively; the sensitivity of the assay was 2.0 μmol/L and its recovery was 93 ± 1.5 %.

To assess intra-individual variance, caused by biological variation and random measurement errors in the urinary and serum values of NO_3_ and NO_2_, two blood and urine samples were collected over a 2-week period, in a sub-sample of the participants (n=41); repeated measurements of urine nitrate may be useful to account for within person variation for validation of NO_3_ intake assessed by food frequency questionnaire (FFQ) (22, 32).

### Dietary assessment

A validated 168-item FFQ was used to assess typical food intakes over the previous year. Trained dietitians, with at least 5 yr of experience in the TLGS survey, asked participants to designate their intake frequency for each food item consumed during the past year on a daily, weekly, or monthly basis. Portion sizes of consumed foods reported in household measures were then converted to grams (33). The validity of the food frequency questionnaire has previously been evaluated by comparing food groups and nutrient values determined from the questionnaire with values estimated from the average of twelve 24-h dietary recall surveys and the reliability has been assessed by comparing energy and nutrient in-takes from two FFQ; Pearson correlation coefficients and intra-class correlation for energy and nutrients showed acceptable agreements between FFQ and twelve 24-h dietary recall surveys, and FFQ1 and FFQ2 (34).

However since Iranian Food Composition Table is incomplete, and has limited data on nutrient content of raw foods and beverages, to analyze foods and beverages for their energy and nutrient content (except NO_3_ and NO_2_), the US Department of Agriculture Food Composition Table was used (4).

### Estimation of NO_3_ and NO_2_ from diet

Food composition values for NO_3_ and NO_2_ were derived from a recent survey conducted on frequently consumed food items among Iranians (25). Briefly, we determined the NO_3_ and NO_2_ contents of 87 food items including grains, legumes, fruits and vegetables, dairy products, meats and processed meats using validated spectrophotometric methods (25). Dietary NO_3_ and NO_2_ intake of the participants, were estimated by multiplying the reported frequency of consumption and portion size of each FFQ item by its NO_3_ and NO_2_ content, and summing values across all food items. In addition to calculating dietary NO_3_ and NO_2_ from all foods, plant- and animal-based NO_3_ and NO_2_ were separately calculated. Additional, we computed contribution of NO_3_ and NO_2_ that came from individual food groups including vegetables, fruits, grains, legumes, dairy, meats and processed meats.

### Estimation of NO_3_ and NO_2_ from drinking water

NO_3_ and NO_2_ intakes from drinking water were derived by estimated daily water intakes of the participants, and measured values of NO_3_ and NO_2_ in tap water, using a spectrophotometric method (35).

### Statistical analysis

Dietary intakes of NO_3_ and NO_2_ were adjusted for total energy intake, according to residuals methods (36). Log-transformed of the variables with non-normal distribution (serum and urine NO_3_ and NO_2_) were used in the analyses. Mean ± standard deviation (SD), or median and inter-quartile range (IQR) was reported for the variables with normal and non-normal distribution, respectively. Pearson correlation and partial correlation test with adjustment of age, sex, body mass index and serum creatinine levels were used to estimate the correlation of dietary NO_3_ and NO_2_ intakes and their urinary and serum levels. The ratio of intra- to inter-individual variance (λ), calculated using the intra class correlation coefficient (ICC), was used for further adjustment of the correlation coefficients (22, 32). The λ was 1.98, 2.12 for urinary and serum NO_3_, 1.95 and 1.85 urinary and serum NO_2_.

All statistical analysis were conducted using SPSS (ver. 16.0, Chicago, IL, USA), and *P*<0.05 were considered significant.

## Results

Mean age of the participants was 41.5±12.4 yr and 31.5% were men. General characteristics of the participants are shown in Table 1. A significant higher serum levels of creatinine was observed in men (104 vs. 88.1 μmol/l, *P*<0.05). There was no significant difference in urinary and serum values of NO_3_ and NO_2_ between men and women.

**Table 1:** Characteristics of the study participants

***Variables***	***Men (n=79)***	***Women (n=172)***	***Total population (n=251)***
Age (yr)	40.8±12.3	41.8±12.5	41.5±12.4
Body mass index (kg/m ^ 2 ^ )	27.0±3.8	27.1±4.8	27.1±4.5
Waist circumference (cm)	94.4±10.0	89.2±12.3	90.9±11.8
Systolic blood pressure (mm Hg)	109±9.5	105±11.5	107.0±11.1
Diastolic blood pressure (mm Hg)	75.7±5.7	72.1±7.1	73.2±6.9
Serum creatinine (μmol/l)	104±9.4	88.1±9.0	93.1±11.8
Urinary NO _ 3 _ (μmol/l)	805 (342–1150)	718 (425–1096)	730 (396–1110)
Urinary NO _ 2 _ (μmol/l)	132 (70.0–140)	130 (69.4–142)	131 (69.4–142)
Serum NO _ 3 _ (μmol/l)	41.9 (21.6–74.5)	43.4 (23.1–65.4)	43.2 (22.6–67.8)
Serum NO _ 2 _ (μmol/l)	15.3 (10.4–19.3)	15.0 (11.8–18.8)	15.1 (11.7–19.0)

Mean ±SD (median and inter-quartile range)

Estimated daily consumption of NO_3_ and NO_2_, from total dietary intake, individual food groups, and drinking water are shown in Table 2. Mean (±SD) intakes of dietary NO_3_ and NO_2_ were 505±160 and 7.7±2.2 mg/d, respectively.

**Table 2:** Estimated daily consumption of nitrate and nitrite in the study population

***Variables***	***Nitrate (mg/d)***		***Nitrite (mg/d)***	
	***Men (n=79)***	***Women (n=172)***	***Men (n=79)***	***Women (n=172)***
Total	532±184	535±221	9.1±3.6	8.3±3.2
Plan-based	498±166	515±216 ^*^	5.5±2.0	5.4±2.2
Animal-based	16.0±11.1	13.7±8.3 ^*^	3.3±2.3	2.8±1.8 ^*^
Legumes	9.5±6.0	9.3±6.2	0.26±0.16	0.25±0.17
Grains	190±86.3	137±71.1 ^*^	2.6±1.6	1.9±1.0 ^*^
Vegetables	242±125	303±173 ^*^	1.3±0.7	1.6±1.1
Fruits	56.4±41.9	65.5±49.5	1.4±1.0	1.7±1.2
Dairy products	9.2±8.8	7.9±6.5	0.7±0.7	0.6±0.5
Meats	5.9±6.0	5.2±4.6	2.1±2.0	1.8±1.6
Processed meats	0.9±1.1	0.6±0.8	0.5±0.4	0.5±0.3
Drinking water	40.2±11.9	34.9±11.0	3.1±0.93	2.7±0.86

Data are mean ±SD

* *P* <0.05 (independent t-test was used)

Mean intakes (±SD) of NO_3_ and NO_2_ from drinking water were 36.6±11.5 and 2.8±0.9 mg/d. There was no significant difference in daily consumption of NO_3_ and NO_2_ from diet and drinking water, between men and women. A significant higher intake of plant-based NO_3_ as well as NO_3_ intakes from vegetables was observed in women (*P*<0.05). In men, compared to women, a higher intake of animal-based NO_3_ and NO_3_ in-take from grains was observed (*P*<0.05).

Pearson correlation coefficients between total intakes and urinary and serum concentrations of NO_3_ and NO_2_ are reported in Table 3. Crude correlation between intake and urinary levels of NO_3_ was 0.39 (*P*=0.013) and 0.19 (*P*=0.026) in men and women, respectively. Adjusting the relationship for age, body mass index and serum creatinine levels, resulted in a higher partial correlation coefficient (*r*=0.42, and *r*=0.29, *P*=0.001 in men and women, respectively). After correction for intra- and inter-individual variance, a stronger correlation between NO_3_ intake and its urinary levels (*r*=0.83, 95% CI=0.56, 0.91, *r*=0.57, 95%CI=0.49–0.67, in men and women, respectively), was observed. A moderate agreement was observed between NO_2_ intake and its urinary levels (*r*=0.27 and 0.29, in men and women). There was no significant association between dietary NO_2_ intake and its serum levels, neither in men (corrected *r*=0.07, 95% CI= −0.08–0.19) or women (corrected *r*=0.09, 95% CI= −0.03, 0.23). Dietary intakes and serum levels of NO_3_ and NO_2_ had a relatively week correlation in both men and women.

**Table 3:** Pearson correlation coefficients between total intakes and urinary and serum concentrations of nitrate and nitrite

***Variables***	***Men***			***Women***						
	***Crude R (P-value)***	***Partial R (P-value) ^1^***	***Corrected R (95% CI) ^2^***	***Crude R (P-value)***	***Partial R (P-value) ^1^***	***Corrected R (95% CI) ^2^***
Nitrate						
Urine	0.39 (0.001)	0.42 (0.002)	0.83 (0.56–0.91)	0.19 (0.026)	0.29 (0.001)	0.57 (0.49, 0.67)
Serum	0.12 (0.35)	0.17 (0.42)	0.36 (0.24–0.46)	0.08 (0.31)	0.09 (0.17)	0.19 (0.07, 0.32)
Nitrite						
Urine	0.13 (0.25)	0.14 (0.30)	0.27 (0.13–0.37)	0.16 (0.055)	0.15 (0.023)	0.29 (0.17, 0.41)
Serum	0.02 (0.88)	0.04 (0.77)	0.07 (−0.08–0.19)	0.08 (0.31)	0.05 (0.48)	0.09 (−0.03, 0.23)

1 Adjusted for age, body mass index and serum creatinine levels.

2 Corrected for intra to inter-individual variance ratio (λ); λ was 1.98, 2.12 for urinary and serum NO _3_ , 1.95 and 1.85 urinary and serum NO _2_

## Discussion

In this study, conducted in the framework of a national population-based cohort, relatively high intakes of NO_3_ and NO_2_ (505±160 and 7.7±2.2 mg/d, respectively) were observed among Iranian population. A great association was observed between dietary NO_3_ intakes with its urinary levels, especially in men, however the association was relatively weak for NO_3_ intakes and its serum levels.

Dietary intakes of NO_3_ and NO_2_ were higher than other reports from different population; the major contributors to nitrate intakes were vegetables including cucumber, green leafy vegetables, lettuce, and tomato, and grains including traditional breads and white rice. The major contributors to NO_2_ intake were lamb and chicken meats, white rice, processed meats, and vegetables such as cucumber and tomato. Compared to other population, grains were the considerable source of plant-based NO_3_ intake in our population, whereas green leafy vegetables such as spinach or other vegetables such as beetroot had a little contribution in our NO_3_ intakes.

Median (inter quartile ranges) intakes of NO_3_ and NO_2_ were reported 309 (215–413) and 1.4 (1.1–1.8) mg/d in the Shanghai Women’s Health Study (37). Median NO_3_ intakes in NIH-AAPR Diet and Health Study were 68.9 and 74.1 mg/d, and NO_2_ intakes were 1.3 and 1.0 mg/d, in men and women, respectively (38). Moreover, our in-takes was approximately twofold of the acceptable daily intake (ADI) values, defined as 3.7 and 0.06 mg/kg body weight for NO_3_ and NO_2_, respectively. In our population, the major contributors to NO_3_ intakes were vegetables (51.2%) and grains (30.7%). Due to a relatively high NO_3_ concentration in our traditional and industrial breads (50.0 mg 100 g^−1^) (25), and high proportion of breads (320 g/d) in dietary pattern of Iranian population (39), NO_3_ exposure from this food group was considerable. We previously reported that mean NO_3_ content of lettuce, potato, radish, and cabbage samples was more than the maximum limits legislated by European countries; moreover, mean NO_2_ contents of fruit samples were also relatively high (25). Dietary intakes of NO_2_ from animal sources accounted for 34.4% of daily mean intake of NO_2_ and the remainder of NO_2_ intake was derived from plant sources. The major contributors to NO_2_ intake were lamb and chicken meats, white rice, processed meats, and vegetables such as cucumber and tomato. High content of NO_3_/NO_2_ in Iranian foods or high intake of NO_3_/NO_2_-containing foods may be contributed in high NO_3_/NO_2_ diet in our population.

A critical overview of current literature shows a limited focus on valid estimation of NO_3_ and NO_2_ intakes (22, 40). Several models, including total diet study model, NO_3_/NO_2_ database model, NO_3_/NO_2_ core food model, the large database model, and a processed meat production model, have been suggested for assessment of dietary exposure of NO_3_ and NO_2_ in epidemiological studies (41). The core food approach, where the core foods are selected on the basis of their NO_3_ and NO_2_ content, seems the best model for estimating NO_3_ and NO_2_ intake from diet (41).

However serum and urine NO_3_ and NO_2_ are affected by endogenous NO production and some factors such as physical activity, using plasma NO_3_ and NO_2_ in epidemiologic studies found to be feasible and the within-person variability is comparable to commonly used biomarkers (42); use of repeated measurements of urine NO_3_ may be capture within person variation and may useful to evaluate the validity of NO_3_ intake assessed by FFQ (43). There was a constant relationship between NO_3_ intake and urinary excretion of NO_3_ and NO_2_ (β=0.6–0.8); increased NO_3_ concentration in the water potentiated the relationship (40). Dietary NO_3_ intake was significantly correlated with its urinary level (*P*=0.01; *r*^2^ = 0.07) following consumption of a diet with high-NO_3_ vegetables (44). A substantial correlation was reported between estimated intake of NO_3_ from FFQ and urinary excretion levels (r=0.59, 95% CI=0.03–0.87), after correction for within person variation and adjustment for sex and body mass index (22); the authors concluded that FFQ assessment of NO_3_ intake may provide valid information on usual NO_3_ intake (22). Cross-checking of FFQ with other dietary assessment method such as 24h- dietary recall, also the performance of FFQ in assessing dietary NO_3_ and NO_2_ intake was comparable to that for many other nutrients; in a recent calibration study in the NIH-AAPR Diet and Health Study, energy-adjusted correlation coefficients between FFQ and 24h-recal based values of dietary nitrate and nitrite, for men and women, respectively were 0.59 and 0.57 for nitrate, and 0.59 and 0.58 for nitrite (38).

Regardless of a good agreement of dietary NO_3_ and its urinary levels, observed in our study, the correlation for serum values was week. This observation can be explained by the fact that ∼ 90% of serum NO_3_ and NO_2_ in fasted state is derived from the L-arginine-NO pathway, and the half-lives of NO_3_ and NO_2_ are only 5–8 h and 20–45 min, respectively (45). Plasma NO_3_ and NO_2_ have observed to be returned to its baseline level within 24 h, after a high-NO_3_ diet (24). Serum NO_3_ and NO_2_ levels may therefore not accurately reflect dietary exposure and seems to be less useful for validation study.

To the best of our knowledge, this is the first estimation of dietary NO_3_ and NO_2_ intakes among Iranian population, based on a comprehensive NO_3_ and NO_2_ database (25) and dietary information derived from a validated FFQ (34), in the framework of a national-cohort on a representative population (27). Our findings should be interpreted considering some strengths and limitations. Use of a validated comprehensive FFQ to assess regular dietary intakes of the participants and estimation of NO_3_/NO_2_ based on measured values in frequently consumed food items among our population (25), compared to other previous studies relied on historic literature values, may fully reflect the accurate NO_3_/NO_2_ exposure from diet. Moreover, all food items identified as major contributors to dietary NO_3_/NO_2_ intakes were on our FFQ.

Our estimation of NO_3_ and NO_2_ however did not capture cooking, peeling, pureeing, fermentation and other food processing methods may affect the NO_3_ and NO_2_ content. Moreover, data on storage time and condition, which can decrease NO3 content of foods, were not available.

## Conclusion

We report here a valid estimation of dietary exposure of NO_3_ and NO_2_ in an Iranian population, for the first time. Dietary NO_3_ and NO_2_ intake, estimated using TLGS FFQ, were greatly correlated with their urinary values, as the accurate surrogate of dietary intake. Using a national database of the NO_3_ and NO_2_ contents of food items along with a valid and reliable FFQ could provide a valid estimation of dietary intakes of NO_3_ and NO_2_ in the target population

## Ethical considerations

Ethical issues (Including plagiarism, informed consent, misconduct, data fabrication and/or falsification, double publication and/or submission, redundancy, etc.) have been completely observed by the authors.
